# Impact of Oral Sebetralstat on Anxiety Associated With Hereditary Angioedema Attacks

**DOI:** 10.1111/cea.70241

**Published:** 2026-03-05

**Authors:** Timothy Craig, Emel Aygören‐Pürsün, Jonathan A. Bernstein, Paula J. Busse, Teresa Caballero, Danny M. Cohn, Mar Guilarte, Henriette Farkas, Douglas H. Jones, Sorena Kiani‐Alikhan, Michael E. Manning, Marcus Maurer, Marc A. Riedl, Sinisa Savic, H. James Wedner, Patrick F. K. Yong, Andrea Zanichelli, Sally van Kooten, Matthew Iverson, Erik Hansen, James Hao, Michael D. Smith, Christopher M. Yea, Paul K. Audhya, William R. Lumry

**Affiliations:** ^1^ Departments of Medicine, Pediatrics, Maternal‐Fetal Medicine, Obstetrics and Gynecology, and Biomedical Sciences Penn State University Hershey Pennsylvania USA; ^2^ Vinmec International Hospital Times City Hanoi Vietnam; ^3^ Vin‐University Hanoi Vietnam; ^4^ University Hospital Frankfurt, Goethe University Frankfurt Frankfurt Germany; ^5^ University of Cincinnati College of Medicine and Bernstein Clinical Research Center Cincinnati Ohio USA; ^6^ Department of Medicine, Division of Clinical Immunology Icahn School of Medicine at Mount Sinai School of Medicine New York New York USA; ^7^ Servicio de Alergia, Hospital Universitario La Paz, Hospital La Paz Health Research Institute (IdiPAZ) Biomedical Research Network on Rare Diseases (CIBERER U754) Madrid Spain; ^8^ Amsterdam University Medical Center, University of Amsterdam Amsterdam the Netherlands; ^9^ Department of Allergy Hospital Universitari Vall D'hebron, Vall D'hebron Research Institute (VHIR) Barcelona Spain; ^10^ Hungarian Angioedema Center of Reference and Excellence, Department of Internal Medicine and Haematology Semmelweis University Budapest Hungary; ^11^ Rocky Mountain Allergy Tanner Clinic Layton Utah USA; ^12^ Division of Infection and Immunity University College London London UK; ^13^ Internal Medicine UA College of Medicine‐Phoenix Phoenix Arizona USA; ^14^ Allergy, Asthma, & Immunology Associates, Ltd Phoenix Arizona USA; ^15^ Institute of Allergology, Charité‐Universitätsmedizin Berlin Corporate Member of Freie Universitätsmedizin Berlin and Humboldt‐Universität Zu Berlin Berlin Germany; ^16^ Fraunhofer Institute for Translational Medicine and Pharmacology (ITMP), Immunology and Allergology Berlin Germany; ^17^ University of California—San Diego La Jolla California USA; ^18^ The Leeds Institute of Rheumatic and Musculoskeletal Medicine University of Leeds Leeds UK; ^19^ Division of Allergy and Immunology, John T. Milliken Department of Medicine Washington University School of Medicine St. Louis Missouri USA; ^20^ Department of Immunology Frimley Health NHS Foundation Trust Frimley UK; ^21^ Operative Unit of Medicine Angioedema Center, IRCCS Policlinico San Donato, Policlinico San Donato Milanese Milan Italy; ^22^ Dipartimento di Scienze Biomediche Per la Salute University of Milan Milan Italy; ^23^ KalVista Pharmaceuticals Salisbury UK; ^24^ KalVista Pharmaceuticals Cambridge Massachusetts USA; ^25^ AARA Research Center Dallas Texas USA

**Keywords:** anxiety, EKTERLY, hereditary angioedema, sebetralstat

## Abstract

**Background:**

People with hereditary angioedema (HAE) experience anxiety from the unpredictable nature of attacks and the burden of parenteral on‐demand therapies, potentially leading to delays or avoidance of treatment. This analysis assessed factors associated with anxiety during attacks and the impact of oral sebetralstat versus placebo on anxiety in the KONFIDENT trial.

**Methods:**

Participants in the randomised, double‐blind, phase 3 KONFIDENT trial (NCT05259917) treated attacks with sebetralstat 300 mg, 600 mg or placebo as early as possible after onset. Anxiety was recorded at treatment administration, every 0.5 h thereafter through 4 h, hourly from 5 to 12 h and every 2 h from 14 to 24 h using an 11‐point modified General Anxiety Numeric Rating Scale (GA‐NRS). Prespecified exploratory endpoints assessed in all attacks and in attacks rated as inducing moderate‐to‐extreme anxiety (GA‐NRS ≥ 4) included cumulative GA‐NRS score and meaningful reduction in anxiety (defined as ≥ 2‐point reduction in GA‐NRS for ≥ 2 consecutive timepoints); least squares mean change from treatment administration in GA‐NRS at 4 and 12 h was also assessed. This study was sponsored by KalVista Pharmaceuticals.

**Results:**

Overall, 115 (44%) attacks were rated as inducing moderate‐to‐extreme anxiety. Female sex, shorter time since HAE diagnosis and greater attack severity were associated with greater anxiety at treatment administration. Reduction in cumulative anxiety after sebetralstat use was significantly greater versus placebo. The time to meaningful reduction in anxiety endpoint showed agreement with time to beginning of symptom relief, reduction in attack severity and complete attack resolution endpoints.

**Conclusion:**

Moderate‐to‐extreme anxiety was common in HAE attacks. Reduction in anxiety was significantly greater in attacks treated with sebetralstat compared with placebo.

## Key Messages

- In the phase 3 KONFIDENT trial, > 40% of hereditary angioedema attacks induced moderate‐to‐extreme anxiety.
- Oral sebetralstat significantly reduced anxiety compared with placebo, especially in attacks rated moderate to extremely anxious.
- The time to reduction in anxiety is strongly associated with the primary and key secondary endpoints.

## Graphical abstract

In the phase 3 KONFIDENT trial of oral sebetralstat for on‐demand treatment of hereditary angioedema attacks, many attacks induced moderate‐to‐extreme anxiety. Sebetralstat reduced anxiety versus placebo; time to reductions agreed with trial endpoints, such as time to beginning of symptom relief. These results suggest that sebetralstat can play a role in alleviating attack‐related anxiety in people living with hereditary angioedema. GA‐NRS, General Anxiety Numeric Rating Scale; HAE, hereditary angioedema.

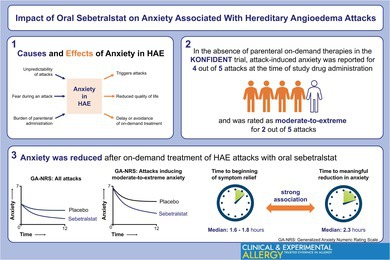

## 1 Introduction

People living with hereditary angioedema (HAE), a genetic disorder that arises from the deficiency or dysfunction of the C1 inhibitor (HAE‐C1INH), experience unpredictable and often debilitating attacks of mucosal and subcutaneous tissue swelling that are associated with substantial psychological disease burden [1, 2, 3, 4, 5, 6]. This psychological burden can include depression, which can affect patients with regard to insomnia and fatigue [6]. It also includes significant anxiety due to the fear of experiencing an attack and fear of the attack worsening or becoming life‐threatening. Anxiety is interconnected with long‐term psychological stress, contributing to a cycle of anxiety and HAE attacks. Studies and patient reports consistently highlight a high prevalence of anxiety in individuals living with HAE, often exceeding rates seen in the general population or in individuals living with other chronic diseases [6, 7]. Through intermediaries such as cytokines, psychological stress may contribute to the activation of the contact system [7, 8, 9], which is insufficiently regulated in HAE.

Surveys of people living with HAE reveal that anxiety may be driven by three overarching stressors: first, the unpredictable timing of the next attack, even among patients receiving long‐term prophylaxis; second, the potential severity of attack symptoms and their impact on daily function (attack‐associated anxiety); third, anxiety associated with treatment, including the potential challenges of injecting or infusing on‐demand medication during an attack, whether treatment will effectively provide symptom relief, and the fear of unnecessarily utilising parenteral on‐demand medication for a mild attack [7, 10, 11]. Approximately one‐half of respondents in the US reported experiencing moderate‐to‐extreme anxiety related to administration of their on‐demand treatment [11]. Moreover, there appeared to be a direct relationship between the level of anxiety and time to treatment in these respondents: 30.0% of respondents who reported no anxiety treated in < 1 h versus 12.1% in those who were extremely anxious [11].

Effective on‐demand therapy that could overcome the burden associated with parenteral therapy may enable improved adherence to treatment guidelines and provide considerable benefit to individuals with HAE. Sebetralstat, an orally administered plasma kallikrein inhibitor, exhibited faster times to beginning of symptom relief, reduction in attack severity and complete attack resolution compared with placebo and was well‐tolerated in patients with HAE‐C1INH in the phase 3 randomised, double‐blind KONFIDENT trial [12]. KONFIDENT was designed to incorporate endpoints that are most relevant to patients [13] and was the first phase 3 trial to include anxiety assessments before and after treatment of attacks. The goal of this analysis from the KONFIDENT trial was to characterise attack‐associated anxiety (in the absence of parenteral on‐demand treatment) and assess the effect of oral sebetralstat compared with placebo on attack‐associated anxiety.

## 2 Methods

### 2.1 Trial Design

The design and inclusion criteria of the phase 3, randomised, placebo‐controlled KONFIDENT trial (NCT05259917) have been previously described [12]. Briefly, participants were ≥ 12 years old with a confirmed diagnosis of HAE‐C1INH and at least 2 documented attacks within 3 months before screening or randomisation. All participants provided written informed consent. Participants receiving long‐term prophylaxis (LTP) must have been on a stable dose and regimen for at least 3 months before screening and throughout the duration of the trial. Attacks of any severity level at any anatomic location were eligible for treatment, except for attacks involving the larynx that were rated as severe or very severe (excluded due to the risk of treating with placebo). Participants were instructed to administer blinded study drug (sebetralstat 300 mg, 600 mg or placebo; according to sequence assignment) as early as possible after attack onset.

To measure the level of attack‐associated anxiety, participants completed an 11‐point modified General Anxiety Numeric Rating Scale (GA‐NRS, ‘How anxious do you feel right now’, Figure S1) from 0 (not at all anxious) to 10 (extremely anxious) at the time of treatment administration and every 0.5 h thereafter through 4 h, hourly from 5 to 12 h and every 2 h from 14 to 24 h.

### 2.2 Anxiety Endpoints

Prespecified exploratory endpoints for anxiety in the KONFIDENT trial were cumulative GA‐NRS score, calculated as the area under the concentration‐time curve from time 0 to 12 h (AUC_0–12_) or AUC from time 0 to 24 h (AUC_0–24_) from administration and the time to meaningful reduction in anxiety (defined as ≥ 2‐point reduction in GA‐NRS score at 2 or more consecutive time points in attacks, with a baseline GA‐NRS score ≥ 2). Least squares mean (LSM) change from baseline at 4 h after treatment administration and 12 h after treatment administration was also assessed. Endpoints were assessed in all attacks and in attacks rated as inducing moderate‐to‐extreme anxiety (i.e., GA‐NRS score ≥ 4) at baseline (time of first study drug administration).

### 2.3 Statistics

Analyses were performed in the full analysis set from the phase 3 KONFIDENT trial, defined as all participants who underwent randomisation and administered the study drug for at least 1 attack according to the assigned sequence [12]. No additional exclusion criteria were applied to the dataset from the parent trial. Coefficients between baseline demographics or attack characteristics and GA‐NRS score at baseline were determined using a generalised linear model with a stepwise model selection and baseline GA‐NRS score set as the dependent variable. Independent variables were age, age group (adolescent or adult) and sex; disease history of anxiety; time since HAE diagnosis and current treatment regimen (on‐demand only or LTP + on‐demand); attack location, attack severity at baseline (categorical), attack number in KONFIDENT (first, second or third attack) and time to first administration of investigational treatment.

Cumulative GA‐NRS score was assessed as the area under the curve (AUC) from treatment administration through to 12 h (AUC_0–12_) and 24 h (AUC_0–24_). LSM change from baseline was analysed with a mixed model for repeated measures including fixed effects of treatment, period, sequence, treatment by analysis time point interaction, and baseline GA‐NRS and random effect for subject nested under sequence. For LSM change from baseline, last observation carried forward (LOCF) was used to impute missing values.

The agreement between ≥ 2‐point reduction in GA‐NRS and beginning of symptom relief was determined using Cohen's kappa analysis. Treatment failure was defined as the use of conventional therapy before reaching each efficacy endpoint; efficacy endpoints were censored at 0 h if the endpoint could not be derived due to missing data. Analyses were not powered to detect statistical significance between treatment groups for GA‐NRS endpoints and were not controlled for multiplicity.

## 3 Results

### 3.1 Participants

In KONFIDENT, 110 participants administered sebetralstat 300 mg, 600 mg or placebo for ≥ 1 attack. The baseline demographics and disease characteristics for these participants have been previously described [12]. Of the 110 participants, 7 (6.4%) were recorded to have a history of anxiety, 1 (0.9%) anxiety disorder, 4 (3.6%) depression and 2 (1.8%) major depression by the study investigator. Three participants (2.7%) were taking concomitant anxiolytics (clonazepam, diazepam, escitalopram oxalate or lorazepam) and 8 participants (7.3%) were taking antidepressants (citalopram, escitalopram oxalate, paroxetine hydrochloride, sertraline, trazodone or vortioxetine hydrobromide).

### 3.2 Characteristics of Attacks Treated With Study Drug

The full analysis set included 264 attacks. The attack characteristics of these attacks have been previously reported [12]. The median GA‐NRS at the time of treatment for the 261 attacks with associated GA‐NRS scores was 3.0 (interquartile range [IQR], 1 to 6). Overall, 115 (44%) of the attacks were rated as inducing moderate‐to‐extreme anxiety (i.e., GA‐NRS ≥ 4) at treatment administration (Table 1 and Table S1). Anxiety records were available for 139 of 141 attacks (98.6%) experienced by 58 participants in Europe and for 77 of 78 attacks (98.7%) treated by 34 participants in North America. The median GA‐NRS at the time of treatment administration was 4.0 (IQR, 1 to 6) in Europe and 3.0 (IQR, 0 to 5) in North America. Seventy attacks (50.4%) and 32 attacks (41.6%) were rated as inducing moderate‐to‐extreme anxiety by participants in Europe and North America, respectively.

**TABLE 1:** Characteristics for attacks inducing moderate‐to‐extreme anxiety.

	Attacks inducing moderate‐to‐extreme anxiety *n* = 115/261 (44%)
Baseline PGI‐S category, *n* (%)^a^	
Mild^a^	38 (33.0)
Moderate	47 (40.9)
Severe/very severe	30 (26.1)
Baseline primary attack location, *n* (%)^b^	
Abdomen	57 (49.6)
Legs/feet	33 (28.7)
Arms/hands	30 (26.1)
Head/face/neck	12 (10.4)
Larynx/throat	4 (3.5)
Genitals	3 (2.6)
Time from onset of attack to first administration, median (IQR), minutes	45 (6–155)
Baseline GA‐NRS category, *n* (%)	
Moderately anxious (4–6)	65 (56.5)
Extremely anxious (7–10)	50 (43.5)

Abbreviations: GA‐NRS, General Anxiety Numeric Rating Scale; IQR, interquartile range; PGI‐S, patient global impression of severity.

^a^ Includes one attack with a PGI‐S rating of “None.”

^b^ Participants with multiple attack locations are counted once in each reported location.

Among demographics, disease characteristics and attack characteristics, female sex, a shorter time since HAE diagnosis, and greater baseline attack severity were significantly associated with greater GA‐NRS scores at treatment administration (Table 2). Age (adults vs. adolescents), disease history of anxiety, attack location, attack number in KONFIDENT (first, second or third), and treatment regimen (on‐demand only vs. on‐demand + LTP) were not significantly associated with greater GA‐NRS scores at the time of treatment administration. To determine whether treatment decisions contributed to the anxiety of an attack, the time from attack recognition to first administration of investigational treatment was also assessed in the model; no significant contribution was identified.

**TABLE 2:** Association of participant demographics, disease characteristics and attack characteristics with anxiety at time of treatment.

Characteristic	*p*
Baseline attack severity^a^	
Severe/very severe versus moderate versus mild/none	< 0.0001
Sex	
Female versus male	0.0021
Time since HAE diagnosis	
Shorter versus longer time	0.0004
Factors not included in the final model	
Age	
Age category (adolescents vs. adults)	
Disease history of anxiety	
Attack location (laryngeal, abdominal only, subcutaneous only, abdominal and subcutaneous)	
Attack location (neck and above, abdominal, other)	
Attack number in KONFIDENT (first, second or third)	
Current treatment regimen (on‐demand only vs. LTP + on‐demand)	
Time to first administration of investigational treatment	

Abbreviations: LTP, long‐term prophylaxis; PGI‐S, Patient Global Impression of Severity.

^a^ Measured as baseline PGI‐S rating.

### 3.3 Impact of Sebetralstat on Attack‐Associated Anxiety

**FIGURE 1:**
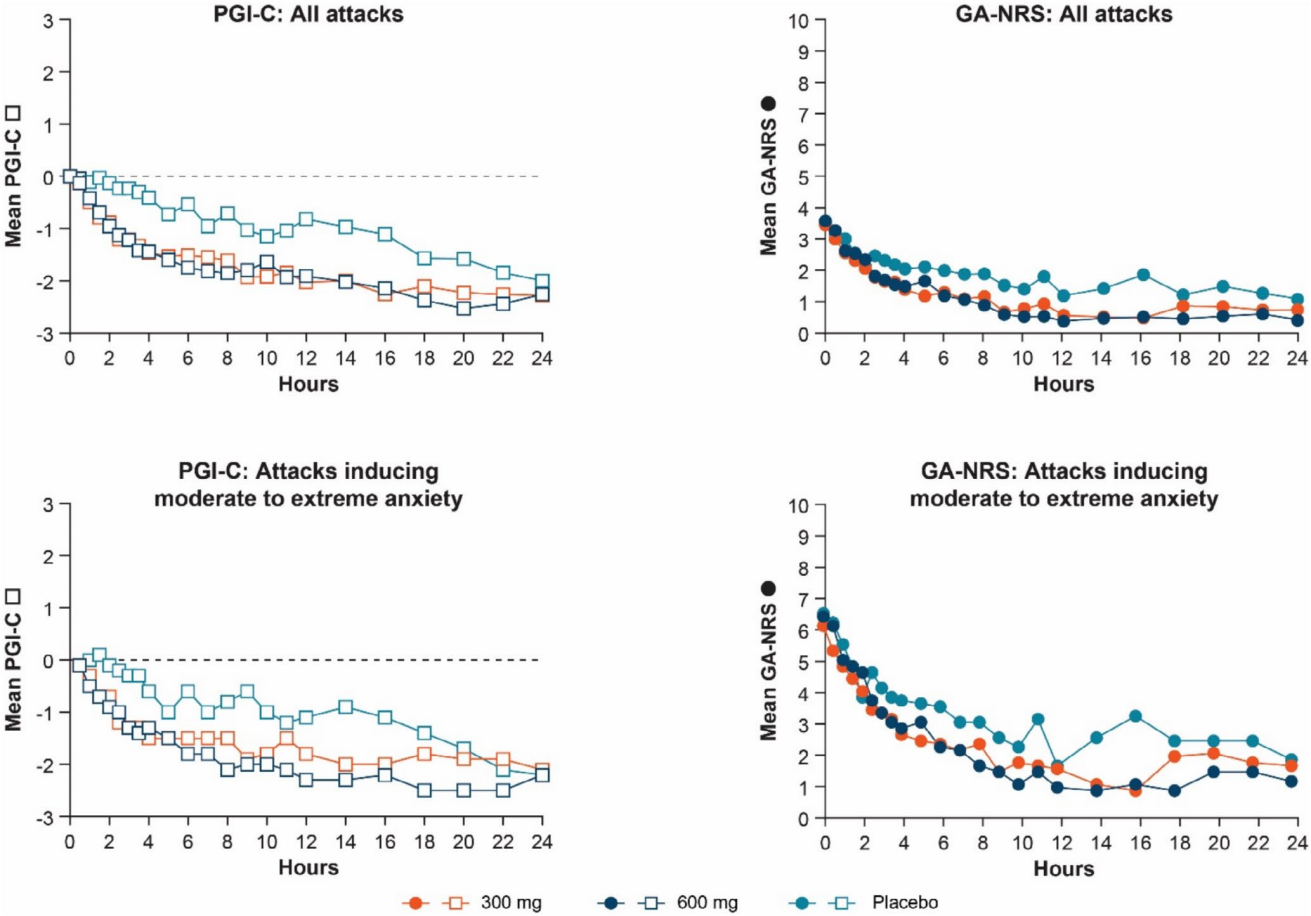
Changes in anxiety over time with sebetralstat 300 mg, sebetralstat 600 mg or placebo. GA‐NRS, General Anxiety Numeric Rating Scale.

Reduction in cumulative GA‐NRS score was greater at 12 h for participants treating attacks with sebetralstat than for those treating with placebo for all attacks (AUC_0‐12_ *p* values of 0.004 with sebetralstat 300 mg and 0.0008 with sebetralstat 600 mg) and attacks that induced moderate‐to‐extreme anxiety (*p* values of 0.0237 and 0.0084). At 24 h, greater reduction in cumulative GA‐NRS score over time was observed with sebetralstat compared with placebo for all attacks (AUC_0‐24_ *p* values of 0.0220 and 0.0012); reduction in GA‐NRS score was significant with 600 mg (*p* = 0.0215) but not with 300 mg (*p* = 0.1807) for attacks that induced moderate‐to‐extreme anxiety (Figure 1).

**FIGURE 2:**
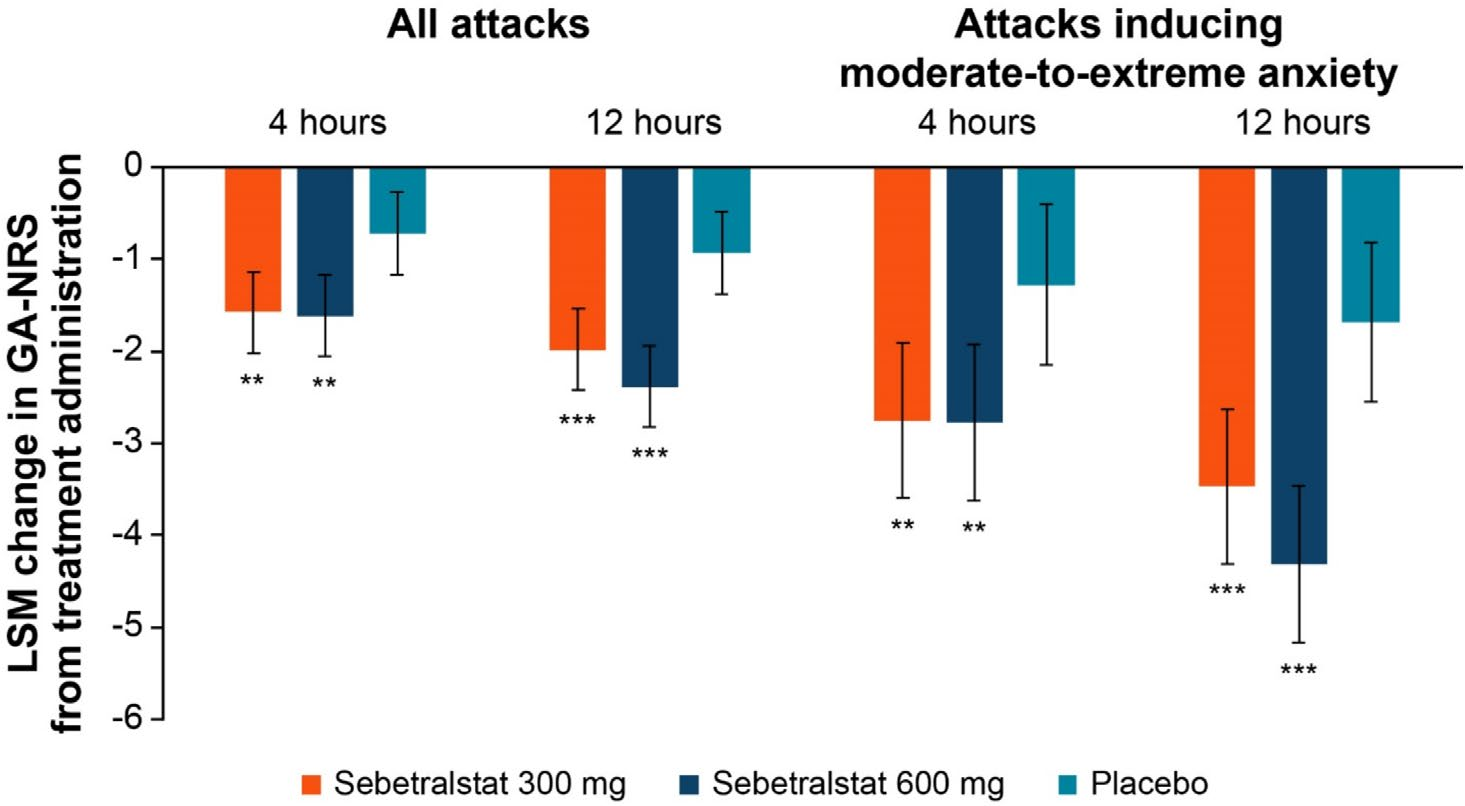
Least squares mean change from treatment administration in GA‐NRS by treatment. GA‐NRS, General Anxiety Numeric Rating Scale; LSM, least squares mean. *p < 0.05, **p < 0.01, ***p < 0.001.

Significantly greater changes in least squares mean (LSM) GA‐NRS ratings from treatment administration were observed at 4 and 12 h for attacks treated with sebetralstat 300 mg or sebetralstat 600 mg compared with placebo (Figure 2). LSM change from treatment administration was more pronounced at 4 and 12 h in attacks that induced moderate‐to‐extreme anxiety at treatment administration compared with all attacks.

**FIGURE 3:**
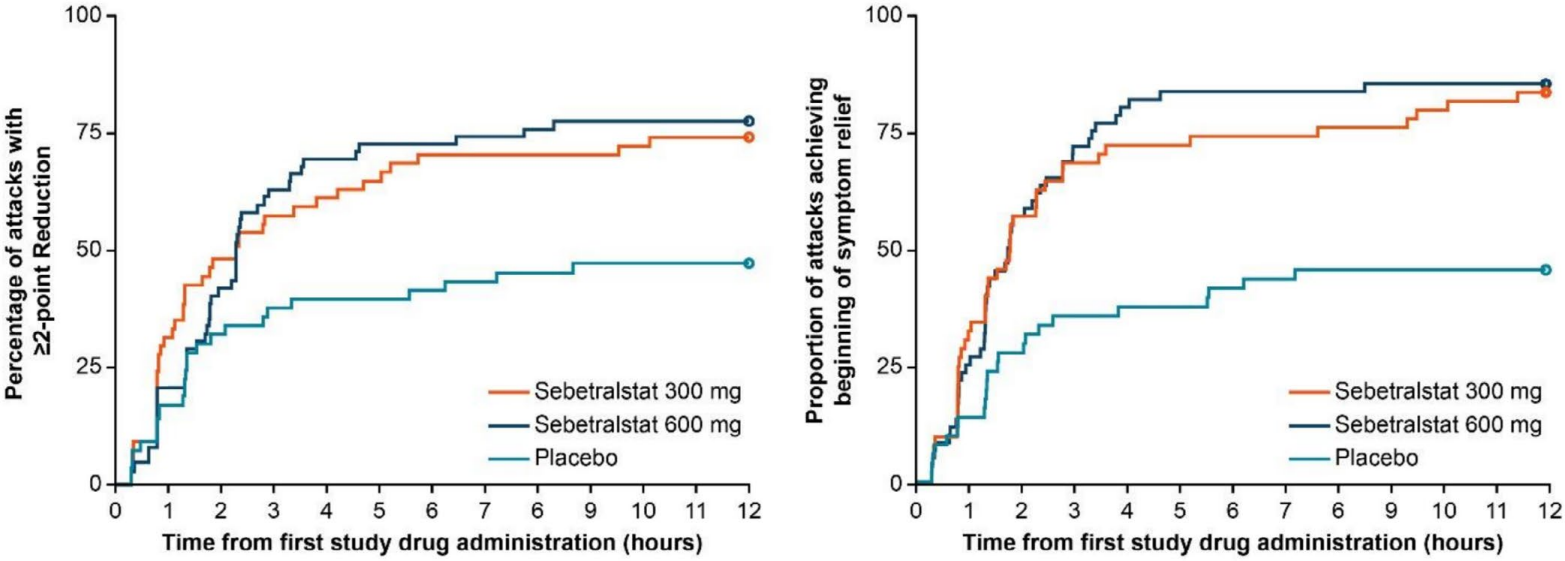
Time to reduction in anxiety within 12 h and time to beginning of symptom relief within 12 h in attacks with GA‐NRS ratings. Time to reduction in anxiety was defined as time at which a ≥ 2‐point reduction in GA‐NRS with a score ≥ 2 at treatment administration was observed within 12 h of the first study drug administration. Attacks with GA‐NRS rating ≥ 2 at treatment administration: Sebetralstat 300 mg, *n* = 56; sebetralstat 600 mg, *n* = 66; placebo, *n* = 54. GA‐NRS, General Anxiety Numeric Rating Scale.

The time to a ≥ 2‐point reduction in anxiety was reached significantly more quickly with either dose of sebetralstat than with placebo (Figure 3). The median time to reduced anxiety was 2.3 h (IQR, 0.8 to > 12) with sebetralstat 300 mg and 2.3 h (IQR, 1.3 to 7.8) with sebetralstat 600 mg versus > 12 h (IQR, 1.3 to > 12) with placebo. The time to a ≥ 2‐point reduction in anxiety across clinically relevant subgroups (baseline attack location, treatment paradigm and age group) was consistent with the overall trial population (Table S2).

### 3.4 Agreement of ≥ 2‐Point Reduction in Anxiety With Achievement of Prespecified Trial Endpoints

Attacks that achieved the primary endpoint in the KONFIDENT trial of beginning of symptom relief also achieved meaningful reduction in anxiety (defined as a ≥ 2‐point reduction in GA‐NRS score from time of treatment administration) within 12 h (Table 3). Similarly, attacks that achieved the key secondary endpoints of reduction in severity within 12 h and complete attack resolution within 24 h also achieved meaningful reduction in anxiety within 12 h. These agreements were consistent across clinically relevant subgroups defined by age (adolescents and adults), attack location (mucosal and subcutaneous attacks) and treatment paradigm (on‐demand treatment only and on‐demand + LTP).

**TABLE 3:** Agreement of ≥ 2‐point reduction in anxiety with achievement of prespecified trial endpoints.

Characteristic	Beginning of symptom relief^a^ within 12 h with reduction in anxiety^b^, ^c^ within 12 h	Reduction in severity^d^ within 12 h with reduction in anxiety^b^, ^c^ within 12 h	Complete attack resolution^e^ within 24 h with reduction in anxiety^b^, ^c^ within 12 h
	Cohen's Kappa (*p*)		
**All attacks**	0.47 (< 0.0001)	0.43 (< 0.0001)	0.37 (< 0.0001)
Attacks inducing moderate to extreme anxiety	0.49 (< 0.0001)	0.43 (< 0.0001)	0.36 (< 0.0001)
**By attack location**			
Mucosal attacks	0.55 (< 0.0001)	0.52 (< 0.0001)	0.47 (< 0.0001)
Abdominal attacks	0.44 (0.0005)	0.50 (< 0.0001)	0.54 (< 0.0001)
Subcutaneous attacks	0.39 (0.0002)	0.37 (< 0.0001)	0.28 (0.0009)
**By treatment paradigm**			
On‐demand only	0.46 (< 0.0001)	0.44 (< 0.0001)	0.35 (< 0.0001)
On‐demand + LTP	0.51 (0.0013)	0.39 (0.0045)	0.44 (0.0037)
**By age group**			
≥ 12 to < 18 years	0.42 (0.0671)	0.60 (0.0045)	0.42 (0.0237)
≥ 18 years	0.48 (< 0.0001)	0.41 (< 0.0001)	0.36 (< 0.0001)

Abbreviations: GA‐NRS, General Anxiety Numeric Rating Scale; LTP, long‐term prophylaxis; PGI‐C, Patient Global Impression of Change; PGI‐S, Patient Global Impression of Severity.

^a^ Defined as a PGI‐C rating of “A Little Better” for ≥ 2 consecutive time points.

^b^ Defined as a ≥ 2‐point reduction in GA‐NRS score for attacks, with a GA‐NRS score ≥ 2 at time of treatment administration.

^c^ In total, 176 attacks with a GA‐NRS score ≥ 2 at treatment administration were included in this analysis.

^d^ Defined as a 1‐point improvement from baseline in PGI‐S score for ≥ 2 consecutive time points.

^e^ Defined as a PGI‐S rating of ‘None’.

## 4 Discussion

Despite the well‐documented physical manifestations of HAE, the significant anxiety experienced by patients appears to be underappreciated in standard care [5, 6, 10, 11]. Current guidelines lack a standardised approach to identifying and managing anxiety, and tools to address anxiety in HAE remain an area of unmet need. Addressing anxiety in a comprehensive manner, alongside managing the physical symptoms of HAE, is vital to improving patients' overall quality of life and empowering them to live more fulfilling lives.

In the KONFIDENT trial, we were able to isolate anxiety related to attack symptoms. Overall, the median level of anxiety at the time of treatment administration for participants in KONFIDENT was consistent with the overarching level of anxiety observed in a survey of patients with HAE living in the United States [10]. Because sebetralstat and placebo were administered orally, anxiety related to carrying and administering injectable treatments was not measured in KONFIDENT. Despite the absence of injectable on‐demand therapy, participants rated more than 40% of attacks in KONFIDENT as inducing moderate‐to‐extreme anxiety at the time of treatment with sebetralstat. Factors that were associated with greater anxiety were female sex, shorter time since receiving an HAE diagnosis, and greater attack severity level at the time of treatment administration. Reduction in anxiety after treatment was significantly greater in attacks treated with sebetralstat compared with placebo, especially among those rated as inducing moderate‐to‐extreme anxiety. This finding was consistent across clinically relevant subgroups, including those defined by attack location, treatment paradigm and age group.

The meaningful improvement in anxiety showed agreement (in Cohen's kappa analysis) with achievement of symptom relief, with a median time to meaningful reduction in anxiety (≥ 2 points) of 2.3 h (with either dose of sebetralstat). Based on medians, participants experienced a meaningful reduction in anxiety approximately 0.5–0.7 h (i.e., 30–42 min) after the beginning of symptom relief [12]. Notably, the median time to reduction in anxiety for attacks treated with placebo was > 12 h. A meaningful reduction in anxiety also showed agreement with the key secondary endpoints: reduction in severity and complete attack resolution.

One described effect of treatment‐related anxiety is the delayed administration of on‐demand therapy [10, 11, 14, 15, 16, 17]. In a recent survey, adults with HAE‐C1INH living in the United States who reported moderate‐to‐extreme anxiety reported a median delay of 1–2 h before administration of treatment [10]. Analysis of an additional survey in adults and adolescents with HAE‐C1INH suggested a relationship between the level of anxiety and the time to treatment, with a mean time to treatment of 5.4 h (SD, 9.4 h) for those individuals who were extremely anxious versus 2.4 h (SD, 2.4 h) in those who were not anxious [11]. This delay could be due to complexities of administering parenteral therapies, including the requirement to transport, store and prepare the treatment; difficulty administering the treatment; the need to interrupt activities or find a private place to administer treatment; and the fear of pain associated with needles or potential injection site reactions. For the latter, 19.4% of adult respondents and 25.0% of adolescent respondents indicated ‘anxiety due to anticipating burning or pain with the injection’. Most adolescents (58.3%) also cited difficulty ‘finding a vein for administration’ as the dominant reason for treatment delay. In contrast, in the absence of injectable treatments in KONFIDENT, the time to treatment was highly similar for all attacks (41 min) [12] including those inducing moderate‐to‐extreme anxiety at the time of treatment (45 min).

The analysis of anxiety reduction with sebetralstat in KONFIDENT has several strengths. These include the randomised, double‐blind, placebo‐controlled design, as well as the inclusion of anxiety reduction as a prespecified endpoint. Furthermore, KONFIDENT included attacks at all severity levels in all anatomical locations, with all treatment decisions left up to the patient. However, assessment of anxiety in a blinded clinical trial setting is also associated with several limitations. First, having to complete the anxiety assessment, alongside multiple other measures, at scheduled intervals for a prolonged period after treatment administration might have affected anxiety levels. To minimise the impact of having to record anxiety, an easy‐to‐complete, 11‐point GA‐NRS was selected. Second, the anxiety of potentially receiving placebo as part of a blinded study may have impacted the anxiety of participants in the KONFIDENT trial. Lastly, assessment of anxiety in a randomised clinical trial may be influenced by participant and investigator selection biases that could either increase baseline anxiety (e.g., if enrollment was driven by distress with current injectable therapies or based on the identification of patients with highest anxiety burdens by investigators) or decrease it (e.g., if enrollment was based on confidence in attack management or if additional education was provided by investigators). However, the pre‐determined exploratory endpoint of area under the GA‐NRS curve through 12 or 24 h was selected to account for the baseline anxiety level and is robust for measuring the overall change in attack‐associated anxiety after the administration of sebetralstat or placebo.

## 5 Conclusion

In conclusion, adults and adolescents with HAE‐C1INH in the KONFIDENT trial experienced significantly reduced attack‐associated anxiety after treatment of attacks with sebetralstat compared with placebo. This reduction was more pronounced in trial participants with attacks that induced moderate‐to‐extreme anxiety. Coupled with the potential to reduce treatment‐related anxiety due to the oral route of administration, these results suggest that sebetralstat can play a role in alleviating attack‐related anxiety that contributes to the burden of disease in people living with HAE.

## Author Contributions

Timothy Craig, Emel Aygören‐Pürsün, Jonathan A. Bernstein, Paula J. Busse, Teresa Caballero, Danny M. Cohn, Mar Guilarte, Henriette Farkas, Douglas H. Jones, Sorena Kiani‐Alikhan, Michael E. Manning, Marcus Maurer, Marc A. Riedl, Sinisa Savic, H. James Wedner, Patrick F.K. Yong, Andrea Zanichelli, Sally van Kooten, Matthew Iverson, Michael D. Smith, Christopher M. Yea, Paul K. Audhya, William R. Lumry: conceptualization, investigation; Emel Aygören‐Pürsün, Jonathan A. Bernstein, Danny M. Cohn, Henriette Farkas, Marcus Maurer, Marc A. Riedl, Andrea Zanichelli, Sally van Kooten, Matthew Iverson, James Hao, Michael D. Smith, Christopher M. Yea, Paul K. Audhya, William R. Lumry: methodology; Matthew Iverson, Erik Hansen, James Hao, Michael D. Smith: formal analysis, data curation; Timothy Craig, Emel Aygören‐Pürsün, Jonathan A. Bernstein, Paula J. Busse, Teresa Caballero, Danny M. Cohn, Mar Guilarte, Henriette Farkas, Douglas H. Jones, Sorena Kiani‐Alikhan, Michael E. Manning, Marcus Maurer, Marc A. Riedl, Sinisa Savic, H. James Wedner, Patrick F.K. Yong, Andrea Zanichelli, Sally van Kooten, Matthew Iverson, Erik Hansen, James Hao, Michael D. Smith, Christopher M. Yea, Paul K. Audhya, William R. Lumry: analysis and/or interpretation of data, writing – review and editing. No artificial intelligence tools were used to generate any portion of the manuscript.

## Funding

This work was supported by KalVista Pharmaceuticals Inc.

## Ethics Statement

The protocol was approved by the institutional review board or ethics committee at each participating institution; initial US approval was provided November 17, 2021, by Advarra (IRB FWA number: 00023875; Pro00058763). The trial was conducted in accordance with the International Conference on Harmonisation Guidelines for Good Clinical Practice, applicable local regulatory requirements and the principles of the Declaration of Helsinki. All the participants provided written informed consent.

## Conflicts of Interest

T.C. has received grants, consulting fees, honoraria and/or served on advisory boards and/or data safety monitoring for KalVista Pharmaceuticals, CSL Behring, GSK, Astria, Takeda, BioMarin Pharmaceutical Inc., BioCryst, Pharming, Ionis Pharmaceuticals, Grifols, Pharvaris, ADARx and Intellia Therapeutics; serves as the director for ACARE International Hereditary Angioedema Center and Alpha‐1 Resource Center; and is a member of the Medical Advisory Board for the HAE‐A. E.A.‐P. has received grants, consulting fees, honoraria and/or served on advisory boards for KalVista Pharmaceuticals, Astria, BioCryst, BioMarin Europe, Centogene, CSL Behring, Intellia Therapeutics, Otsuka, Pharming Technologies, Pharvaris and Takeda/Shire. J.A.B. has received grants and/or honoraria from KalVista Pharmaceuticals, BioCryst, BioMarin, CSL Behring, Intellia Therapeutics, Ionis Pharmaceuticals, Pharming, Pharvaris and Takeda/Shire and serves as the immediate past president of the American Academy of Allergy, Asthma & Immunology (AAAAI). P.J.B. has received consulting fees from KalVista Pharmaceuticals, Adarx, Astria, BioCryst, BioMarin, CSL Behring, CVS Specialty, Novartis, Pharvaris, Regeneron and Takeda; has received payment for expert testimony from Hinkley Allen; and has a leadership role in the Hereditary Angioedema Association (HAEA). T.C. has received grants and royalties and/or licences paid to the institution, consulting fees, honoraria, medical writing support, funded clinical trials, article processing charges, meeting/travel support, patient medication (Hospital Universitario La Paz), educational course sponsorship and/or served on advisory boards for KalVista Pharmaceuticals, Takeda, CSL Behring, AEDAF, Ionis Pharmaceuticals, Pharming, BioCryst, Novartis, Astria, Pharvaris and Otsuka Pharmaceuticals and is a developer of the HAE‐QoL patient reported outcomes questionnaire. D.M.C. has received consulting fees paid to the institution, honoraria paid to the institution, medical writing support, meeting/travel support, research support; has served on advisory boards from KalVista Pharmaceuticals, Astria, BioCryst, CSL Behring, Intellia Therapeutics, Ionis Pharmaceuticals, Otsuka, Pharvaris and Takeda; and has had a leadership role in the HAEi Medical Advisory panel for Central Eastern Europe and Benelux. M.G. has received grants, honoraria, meeting/travel support and has served on advisory boards for KalVista Pharmaceuticals, BioCryst, CSL Behring, Takeda, Novartis, Pharvaris and Otsuka Pharmaceuticals. H.F. has received grants paid to the institution, honoraria, medical writing support and meeting/travel support; has served on advisory boards for KalVista Pharmaceuticals, Astria, BioCryst, CSL Behring, Intellia, Ono Pharmaceutical, Pharming, Pharvaris and Takeda; and has had a leadership role on the Angioedema Centers of Reference and Excellence (ACARE) Steering Committee. D.H.J. has received consulting fees, served as a speaker and/or served on advisory boards for KalVista Pharmaceuticals, Amerimmune Allergy Testing Inc., AstraZeneca, BioCryst Pharmaceuticals, Pharming, Pharvaris, Regeneron/Sanofi, Shire/Takeda and Zurvita Corporation. S.K.‐A. was the chief and principal investigator for studies and in receipt of honorarium for consulting work and advisory boards organised by KalVista Pharmaceuticals, Astria, BioCryst, Biotest, CSL Behring, Ionis, Otsuka Pharmaceuticals, Pharvaris and Shire/Takeda. M.E.M. has received grants, consulting fees, honoraria, clinical trial support, medical writing support, article processing charges and meeting/travel support and has served on advisory boards and/or data safety monitoring for KalVista Pharmaceuticals, BioCryst, CSL Behring, Ionis Pharmaceuticals, Pharvaris, Pharming, Takeda, Astria, BioMarin Pharmaceutical Inc. and Intellia Therapeutics. M.M. has received grants, consulting fees and honoraria and/or served on advisory boards for KalVista Pharmaceuticals, Allakos, Alexion, Almirall, Alvotech, Amgen, Aquestive, Arcensus, argenX, AstraZeneca, Astria, BioCryst, Blueprint, Celldex, Celltrion, Clinuvel, Cogent, CSL Behring, Escient, Evoemmune, Excellergy, GSK, Incyte, Jasper, Kashiv, Kyowa Kirin, Leo Pharma, Lilly, Menarini, Mitsubishi Tanabe Pharma, Moxie, Noucor, Novartis, Orion Biotechnology, Pharvaris, Resonance Medicine, Sanofi/Regeneron, Santa Ana Bio, Septerna, Servier, Takeda, Teva, Third HarmonicBio, Valenza Bio, Vitalli Bio, Yuhan Corporation and Zurabio and has had a leadership role as treasurer at Global Allergy and Asthma European Network (GA^2^LEN). M.A.R. has received grants, consulting fees and funded clinical trial from KalVista Pharmaceuticals, BioCryst, BioMarin, CSL Behring, Ionis Pharmaceuticals, Pharvaris, Takeda, Astria, Celldex, Cycle Pharma, Grifols, Intellia Therapeutics, Pfizer, Pharming and Sanofi/Regeneron. S.S. has received grants, consulting fees, honoraria and/or meeting/travel support from KalVista Pharmaceuticals, CSL Behring, Novartis, SOBI and Takeda and has a leadership position in the British Society for Immunology Clinical Immunology Professional Network (BSI‐CIPN). P.F.K.Y. has received consulting fees, honoraria, medical writing support and meeting/travel support and has served on advisory boards for KalVista Pharmaceuticals, CSL Behring, BioCryst, Astria Therapeutics, Pharming, Pharvaris, Otsuka Pharmaceuticals and Takeda and has had a leadership role in the BSI‐CIPN Steering Committee. A.Z. has received honoraria and meeting/travel support and/or served on advisory boards for KalVista Pharmaceuticals, BioCryst, CSL Behring, Pharvaris and Takeda. W.R.L. has received consulting fees, grants and/or research support from KalVista Pharmaceuticals, Astria, BioCryst, BioMarin, CSL Behring, Express Scripts/CVS, Fresenius Kabi, Intellia, Magellan, Optum, Pharming, Pharvaris, Shire/Takeda, Optinose, Grifols, AstraZeneca, Sanofi/Regeneron, GSK, Ionis Pharmaceuticals and Teva and has board membership with US Hereditary Angioedema Association Medical Advisory Board and DFW Metroplex Allergy Society. S.K., M.I., E.H., J.H., M.D.S., C.M.Y. and P.K.A. are employees and shareholders of KalVista Pharmaceuticals. H.J.W. has nothing to disclose.

## Supporting information


**Figure S1:** General Anxiety Numeric Rating Scale.
**Table S1:** Proportion of attacks inducing moderate‐to‐extreme anxiety by baseline attack characteristics.
**Table S2:** Time to meaningful reduction in anxiety within 12 h by study drug and clinical subgroups.

## Data Availability

KalVista accepts requests from qualified researchers who wish to access clinical trial data and associated information, such as Clinical Study Reports (CSRs) with appropriately redacted appendices to protect participant privacy. Please direct your inquiry to dsp@kalvista.com for more details.
