# Supplementary material for: Impact of Oral Sebetralstat on Anxiety Associated With Hereditary Angioedema Attacks
Source: Clin Exp Allergy. 2026 Mar 5;56(7):739–47. doi: 10.1111/cea.70241 (PMC13327167; doi:10.1111/cea.70241)

**Impact of Oral Sebetralstat on Anxiety Associated With Hereditary Angioedema Attacks**

Timothy Craig,^1,2,3^ Emel Aygören-Pürsün,^4^ Jonathan A. Bernstein,^5^ Paula J. Busse,^6^ Teresa Caballero,^7^ Danny M. Cohn,^8^ Mar Guilarte,^9^ Henriette Farkas,^10^ Douglas H. Jones,^11^ Sorena Kiani-Alikhan,^12^ Michael E. Manning,^13,14^ Marcus Maurer,^15,16^ Marc A. Riedl,^17^ Sinisa Savic,^18^ H. James Wedner,^19^ Patrick F. K. Yong,^20^ Andrea Zanichelli,^21,22^ Sally van Kooten,^23,24^ Matthew Iverson,^23,24^ Erik Hansen,^23,24^ James Hao,^23,24^ Michael D. Smith,^23,24^ Christopher M. Yea,^23,24^ Paul K. Audhya,^23,24^ William R. Lumry^25^

^1^Departments of Medicine, Pediatrics, Maternal-Fetal Medicine, Obstetrics and Gynecology, and Biomedical Sciences, Penn State University, Hershey, Pennsylvania, USA; ^2^Vinmec International Hospital, Times City, Hanoi, Vietnam; ^3^Vin-University, Hanoi, Vietnam; ^4^University Hospital Frankfurt, Goethe University Frankfurt, Frankfurt, Germany; ^5^University of Cincinnati College of Medicine and Bernstein Clinical Research Center, Cincinnati, Ohio, USA; ^6^Department of Medicine, Division of Clinical Immunology, Icahn School of Medicine at Mount Sinai School of Medicine, New York, New York, USA; ^7^Servicio de Alergia, Hospital Universitario La Paz, Hospital La Paz Health Research Institute (IdiPAZ), Biomedical Research Network on Rare Diseases (CIBERER U754), Madrid, Spain; ^8^Amsterdam University Medical Center, University of Amsterdam, Amsterdam, the Netherlands; ^9^Department of Allergy, Hospital Universitari Vall d’Hebron, Vall d’Hebron Research Institute (VHIR), Barcelona, Spain; ^10^Hungarian Angioedema Center of Reference and Excellence, Department of Internal Medicine and Haematology, Semmelweis University, Budapest, Hungary; ^11^Rocky Mountain Allergy, Tanner Clinic, Layton, Utah, USA; ^12^Division of Infection and Immunity, University College London, London, UK; ^13^Internal Medicine, UA College of Medicine-Phoenix, Phoenix, Arizona, USA; ^14^Allergy, Asthma, & Immunology Associates, Ltd. Phoenix, Arizona, USA; ^15^Institute of Allergology, Charité-Universitätsmedizin Berlin, corporate member of Freie Universitätsmedizin Berlin and Humboldt-Universität zu Berlin, Berlin, Germany; ^16^Fraunhofer Institute for Translational Medicine and Pharmacology (ITMP), Immunology and Allergology, Berlin, Germany; ^17^University of California - San Diego, La Jolla, California, USA; ^18^The Leeds Institute of Rheumatic and Musculoskeletal Medicine, University of Leeds, Leeds, UK; ^19^Division of Allergy and Immunology, John T. Milliken Department of Medicine, Washington University School of Medicine, St. Louis, Missouri, USA; ^20^Department of Immunology, Frimley Health NHS Foundation Trust, Frimley, UK; ^21^Operative Unit of Medicine, Angioedema Center, IRCCS Policlinico San Donato, Policlinico San Donato Milanese, Milan, Italy; ^22^Dipartimento di Scienze Biomediche per la Salute, University of Milan, Milan, Italy; ^23^KalVista Pharmaceuticals, Salisbury, United Kingdom; ^24^KalVista Pharmaceuticals, Cambridge, Massachusetts, USA; ^25^AARA Research Center, Dallas, Texas, USA

**Correspondence:**

Timothy Craig (tcraig@pennstatehealth.psu.edu)

**Funding Information**

KalVista Pharmaceuticals, Inc

**Supplementary Table S1.** Proportion of attacks inducing moderate-to-extreme anxiety by baseline attack characteristics

|  | **All attacks,  n (%)** | **Attacks inducing  moderate-to-extreme anxiety,  n/n (% of subgroup)** |
| --- | --- | --- |
| Baseline PGI-S category^a^ |  |  |
| Mild | 115^b^ | 38/115 (33.0)^c^ |
| Moderate | 102 | 47/102 (46.1) |
| Severe/very severe | 45 | 30/45 (66.7) |
| Baseline primary attack location^a^ |  |  |
| Abdomen | 114 | 57/114 (50.0) |
| Legs/feet | 62 | 33/62 (53.2) |
| Arms/hands | 76 | 30/76 (39.5) |
| Head/face/neck | 29 | 12/29 (41.4) |
| Larynx/throat | 8 | 4/8 (50.0) |
| Genitals | 9 | 3/9 (33.3) |

Abbreviation: PGI-S, Patient Global Impression of Severity.

^a^The PGI-S rating and attack location at baseline are missing for 2 attacks.

^b^Including 2 attacks with a baseline PGI-S rating of “None.”

^c^Including 1 attack with a baseline PGI-S rating of “None.”

**Supplemental Table 2.** Time to Meaningful Reduction in Anxiety Within 12 Hours by Study Drug and Clinical Subgroups

|  | Sebetralstat 300 mg | | Sebetralstat 600 mg | | Placebo | |
| --- | --- | --- | --- | --- | --- | --- |
|  | Number of attacks, n | Median (IQR) | Number of attacks, n | Median (IQR) | Number of attacks, n | Median (IQR) |
| All Attacks | 56 | 2.3 (0.8-10.1) | 66 | 2.3 (1.3-5.5) | 54 | >12 (1.3->12) |
| Attacks that induced  moderate-to-extreme anxiety | 39 | 1.8 (0.8-10.1) | 40 | 2.3 (1.3-5.5) | 36 | 6.2 (1.3->12) |
| Attack location |  |  |  |  |  |  |
| Mucosal attacks | 24 | 2.5 (0.8->12) | 36 | 1.9 (0.8-7.8) | 26 | >12 (2.1->12) |
| Abdominal attacks | 17 | 2.0 (0.8->12) | 27 | 2.3 (0.8-7.8) | 19 | >12 (1.3->12) |
| Subcutaneous attacks | 32 | 1.8 (0.8-5.7) | 30 | 2.3 (1.7-4.6) | 28 | >12 (1.3->12) |
| By treatment paradigm |  |  |  |  |  |  |
| On-demand only | 42 | 1.6 (0.8-9.5) | 52 | 2.3 (1.3-5.5) | 43 | >12 (1.3->12) |
| On-demand + LTP | 14 | 3.3 (0.8->12) | 14 | 2.3 (1.3-5.9) | 11 | 6.2 (1.3->12) |
| By age group |  |  |  |  |  |  |
| ≥12 to <18 years | 4 | >12 (3.2->12) | 7 | 2.8 (0.8->12) | 8 | 5.8 (0.6->12) |
| ≥18 years | 52 | 1.8 (0.8-9.5) | 59 | 2.3 (1.3-4.6) | 46 | >12 (1.3->12) |

Abbreviation: LTP, long-term prophylaxis.

**Supplementary Figure S1.** General Anxiety Numeric Rating Scale


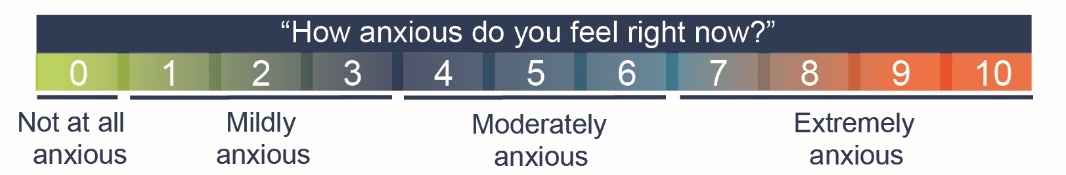

Supplement: Supplementary file 1 — Figure S1: General Anxiety Numeric Rating Scale. Table S1: Proportion of attacks inducing moderate‐to‐extreme anxiety by baseline attack characteristics. Table S2: Time to meaningful reduction in anxiety within 12 h by study drug and clinical subgroups. [file CEA-56-739-s001.docx]
